# Local Sympathetic Denervation of Femoral Artery in a Rabbit Model by Using 6-Hydroxydopamine *In Situ*

**DOI:** 10.1155/2014/874947

**Published:** 2014-06-30

**Authors:** Yufei Jin, Junjun Fan, Fuhang Li, Long Bi, Guoxian Pei

**Affiliations:** Department of Orthopaedics, Xijing Hospital, Fourth Military Medical University, Xi'an 710032, China

## Abstract

Both artery bundle and sympathetic nerve were involved with the metabolism of bone tissues. Whether the enhancing effects of artery bundle result from its accompanying sympathetic nerve or blood supply is still unknown. There is no ideal sympathetic nerve-inhibited method for the *in situ* denervation of artery bundle. Therefore, we dipped the femoral artery in the 6-hydroxydopamine (6-OHDA) locally and observed its effect. Compared with control group, the *in situ* treatment of 6-OHDA did not damage the normal structure of vascular bundle indicated by hematoxylin-eosin (HE) staining. However, the functions of sympathetic nerve was completely inhibited for more than 2 weeks, and only a few function of sympathetic nerve resumed 4 weeks later, evidenced by glyoxylic acid staining and the expression of tyrosine hydroxylase (TH) and nerve peptide Y (NPY). Thus, 6-OHDA is promising as an ideal reagent for the local denervation of sympathetic nerve from artery system.

## 1. Introduction

Both blood supply and nerve innervation play an important role in the development, growth, and maturity of tissues and organs. Furthermore, they are also essential for bone regeneration. The vascularization and neurotization are very important for the clinical application of bone tissue engineering [1]. Some researchers proved that tissue-engineered bone with vascular bundle implanted could significantly improve the effect of bone regeneration [2]. However, the vascular bundle used in the experiment was rich in sympathetic nerve fiber. Sympathetic nerve has been proven to be involved in the metabolism and regeneration of bone tissues [3]. Whether the enhancing effects of artery bundle result from its accompanying sympathetic nerve or blood supply is still unknown. So it is very important to find a sympathetic nerve-inhibited method for the* in situ* denervation of artery bundle. 6-OHDA is the most common medicament used for the chemical denervation of sympathetic nerve in many researches, but there is still no ideal model of arterial sympathetic denervation* in situ*. In this study, we try to establish a rabbit model of local sympathetic denervation of femoral artery by using 6-OHDA* in situ* and to find an effective method to eliminate the sympathetic nerve from the arterial vessel wall temporarily.

## 2. Materials and Methods 

### 2.1. Animals

All the experimental protocols were consistent with the* NIH Guidelines for the Care and Use of Laboratory Animals. *Totally, 16 male New Zealand Rabbits aged three months, weighing 2.5 to 3.0 kg, were randomly divided into three experimental groups and one control group with four rabbits in each group. The experimental groups were divided into 7th-day group, 14th-day group, and 28th-day group according to different time points after the treatment of 6-OHDA. The control group was not treated with 6-OHDA.

### 2.2. Preparation of Reagents

Krebs buffer was prepared according to the previous research [4]. For the experimental groups, 0.1 mg/mL 6-OHDA and 0.1% ascorbic acid were added into the Krebs buffer. But for the control group, only 0.1% ascorbic acid was added into Krebs buffer [5]. And 3% pentobarbital solution was prepared at the same time.

### 2.3. Operation of Animals

All the rabbits were weighed before operation and were anesthetized with the intravenous injection of pentobarbital at a dose of 30 mL/kg. The rabbits were put in the supine position with the knee inflexion between 155° and 165°. A longitudinal incision was made along the direction of femoral artery. The femoral artery, femoral vein, femoral nerve, and muscle tissues were found and dissociated under the microscope. All the small vessel branches of femoral artery and femoral vein were ligatured and cut off without damaging the vessel wall. After the dissociation, the surface of femoral artery was wrapped by the sterile pads. Then an asepsis plastic diaphragm with the size of 5 cm in length and 4 cm in width was placed under the femoral artery to separate the femoral artery from veins, nerve, and other tissues. The prepared drug solution was continually dripped on the pads and the femoral artery was soaked in the drug solution for 4 hours. At the end of the experiment, the pads and diaphragm were removed, and the incision was closed with 3–0 suture. For the first three days after operation, intramuscular injection of 20000–30000 u/kg/day penicillin was used for the prevention of infection.

### 2.4. HE Staining

The femoral artery tissues of control group and each experimental group were harvested, fixed in 1% paraformaldehyde, and embedded in paraffin. 10 sections were prepared with a 5 *μ*m thickness and stained with hematoxylin and eosin (HE) in the middle of the femoral artery. The specimens were observed under the light microscope (Nikon Eclipse 90I).

### 2.5. Fluorescent Histochemical Staining

The fresh femoral vascular tissues from control group and each experimental group were collected and the surrounding adipose tissues were removed, and then the vascular tissues were rinsed with PBS (PH = 0.1 M, 0.1). Then, the blood vessels were split along the vertical axis and put into the optical coherence tomography (OCT) with the adventitial side up, liquid nitrogen freezing, and serial frozen section. The thickness of section was 15–20 *μ*m. Before staining, 20 mL glyoxylic acid solution was freshly prepared. Staining procedures were based on the previous methods [6]. After the slices were dried with a blower-dryer, 2% w/v glyoxylic acid in 0.1 M phosphate buffer (pH 7.2) was added by drop-wise addition at room temperature for 5 min and repeated 3 times. Then the slices were dried and put into an oven of 100°C for 5 min. After that, the slices were covered with glycerin in the glass slide and reheated to 80°C for 2 min in the oven. In the blank group, the glyoxylic acid was replaced with PBS. After staining, specimens were observed under the fluorescence microscope (Nikon Eclipse 90I). Image J processing software was applied to analyze the OD values of fluorescence.

### 2.6. Western Blotting Assay

To detect the protein expression of TH and NPY in the femoral artery, the femoral artery tissues from each experimental group and control group were collected and stored at −80°C. Proteins were extracted from artery lysate and denatured in SDS as the method described previously [7]. Then, 30 *μ*g proteins were used in each gel electrophoresis lane of 10% SDS-PAGE. The proteins were transferred to the Invitrolon polyvinylidene fluoride (PVDF) membrane (Invitrogen, US), and then nonspecific binding was blocked for 2 hours in the blocking buffer. The membranes were incubated with monoclonal Anti-TH (Abcam, UK) and polyclonal Anti-NPY (Sigma, US) overnight at 4°C. The blots were washed with Tris-buffered saline Tween-20 and then incubated with horseradish peroxidase-conjugated secondary antibodies (ZSGB-BIO, CN) for 1 hour at room temperature. By using chemiluminescence reaction, the antibodies were revealed. The Alphalmager Gel Imaging System (Alpha Innotech, CA) was used to quantify the expression of proteins and the Image J processing software was used to quantify the band intensity.

### 2.7. Statistical Analysis

Data were presented as mean ± standard deviation (SD). Intergroup comparison was analyzed with one-way analysis of variance (ANOVA); the pair-wise comparison was analyzed with LSD test. *P* values less than 0.05 were considered statistically significant.

## 3. Results

### 3.1. Histopathological Staining

**Figure 1:**
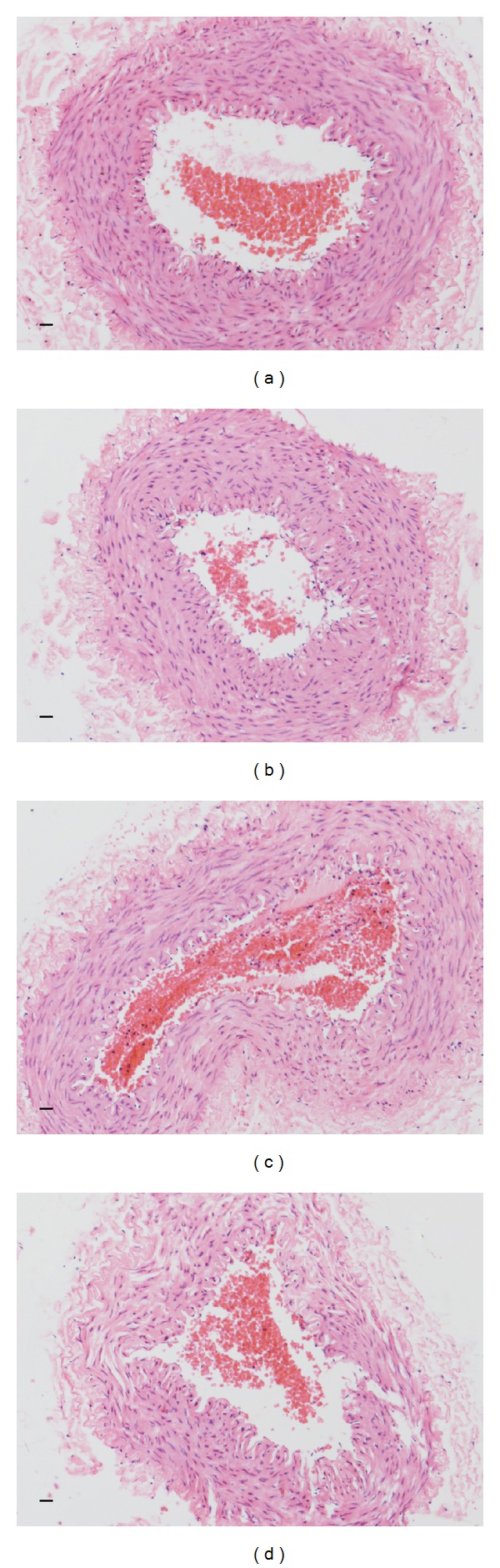
Hematoxylin-eosin (HE) staining of femoral arteries ((a) control group, (b) 7th-day group, (c) 14th-day group, and (d) 28th-day group.* Scale bars = *50 *μ*m).

In the experimental groups, the structure of femoral artery such as medial membrane and outer membrane was as intact as in the control group. Furthermore, the endothelial cells and the morphological structure of vessel wall were completely maintained. There was no significant difference between each experiment group and control group (Figure 1).

### 3.2. Fluorescent Histochemical Staining

**Figure 2:**
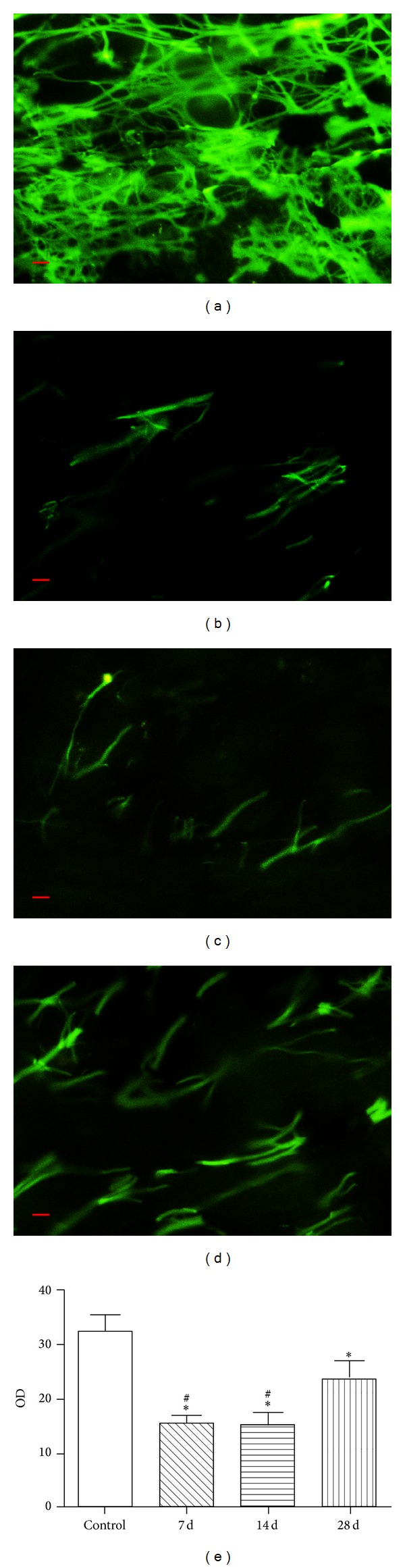
Glyoxylic acid staining of femoral arteries. The yellow-green fluorescence represents the presence of monoamine substance. (a) Control group; (b) 7th-day group; (c) 14th-day group; (d) 28th-day group; (e) OD values of different groups (∗*P* < 0.05: significant difference from the control group. ^#^ *P* < 0.05: significant difference from the 28th-day group.* Scale bars = *20 *μ*m).

Glyoxylic acid stimulated monoamine substance to emit yellow-green fluorescence colors, and a large number of sympathetic nerves distributed in the blood vessel wall with the reticular and radialized pattern could be found in the femoral artery of control group (Figure 2). However, the sympathetic nerve distributed in the blood vessel wall was almost invisible in the 7th-day group (Figure 2(b)) and 14th-day group (Figure 2(c)). And there were a few regenerative nerve fibers shown in the femoral arteries on postoperative day 28 (Figure 2(d)). However the quantity of new nerves was much less than the quantity of sympathetic nerves in the normal vascular wall (Figure 2(a)). By analyzing the fluorescence OD value, the OD value of the control group was 32.40 ± 7.42, and the OD value of  7th-day group, 14th-day group, and 28th-day group was 15.30 ± 3.79, 15.20 ± 2.18, and 23.65 ± 8.20, respectively. The OD value of each experiment group was significantly decreased compared with the control group (*P* < 0.05). And the OD value of the 28th-day group was increased compared with the other two experiment groups (*P* < 0.05) (Figure 2(e)).

### 3.3. TH and NPY Expression

**Figure 3:**
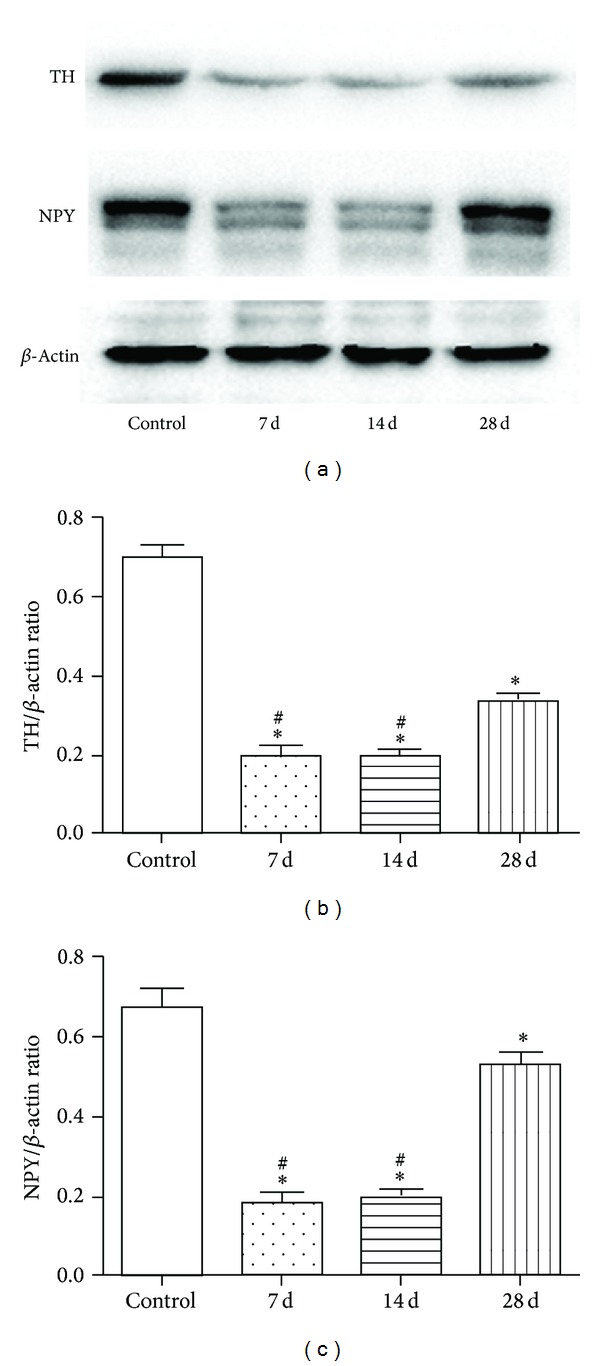
Expression of tyrosine hydroxylase (TH) and nerve peptide Y (NPY) in control group and experimental groups. (a) Western blotting result of TH and NPY in control and experimental groups; (b) expression of TH was shown as the ratio of TH to *β*-actin in all groups; (c) expression of NPY was shown as the ratio of TH to *β*-actin in all groups. (∗*P* < 0.05: significant difference from the control group. ^#^ *P* < 0.05: significant difference from the 28th-day group.)

By using the method of western blot, both TH and NPY expressions in each experimental group were significantly decreased compared with the control group (all *P* < 0.05) (Figure 3(a)). The TH and NPY expression of all three experimental groups had the same tendency and was significantly decreased at the 7th and 14th day after operation (all *P* < 0.05). Then the expression amount was increased on the 28th day. However, the quantity was much less than the normal level (*P* < 0.05). By Western blotting analysis, the expression of TH in each experimental group was significantly lower than that in control group (all *P* < 0.05) (Figure 3(b)). Meanwhile, The NPY expression in each experimental group was significantly lower than that in the control group (all *P* < 0.05) (Figure 3(c)).

## 4. Discussion

6-OHDA is the most common medicament used for the chemical denervation of sympathetic nerve and is also a kind of neurotoxin which can selectively damage the neuron of dopamine and norepinephrine. As a kind of nerve agents, 6-OHDA can selectively destroy the sympathetic nerve ending and not pass through the blood-brain barrier when used topically. So it is now recognized as the ideal reagent of chemical sympathectomy [8]. But 6-OHDA is unstable and easy to be oxidized, so the solution should be freshly prepared in the experiment. According to the previously reported literature, 6-OHDA was injected into the nigra-striatum system of the brain and caused the damage of artery sympathetic nerve. And this method was proved to be stable for the establishment of Parkinson animal model [9]. In recent years, there are also other methods to establish the animal model of sympathetic nerve denervation with the 6-OHDA given by intravenous injection, subcutaneous injection, and intraperitoneal injection [10]. However, those methods have some disadvantages such as extra damage to the systemic nervous system or most of the sympathetic nerve system. Suzuki reported a local denervation method for the treatment of cerebrovascular Moyamoya disease by using bilateral cervical perivascular sympathectomy (PVS) [11]. But the native structure of vessel wall was damaged by this method. Then Warland reported a successful model of vascular sympathetic denervation* in vitro* by soaking the rabbit saphenous artery with 6-OHDA for 4 hours and the structure of vessel wall was preserved [12]. On the basis of their research, we successfully provided an animal model of vascular sympathetic denervation* in situ* using the 6-OHDA. In our study, effective sympathetic denervation of femoral artery was performed by locally using 6-OHDA without damage to the vessel wall as shown in HE staining. We also found that our method did not permanently remove the sympathetic nerve from femoral artery and, thus, did not damage the normal function of blood vessels when used to repair the bone defect.

A large number of sympathetic nerve fibers were distributed in the normal arterial wall and could be observed as reticular and radialized pattern by glyoxylic acid staining [13]. Li established the mesenteric vascular model of sympathetic denervation by using the celiac ganglionectomy (CGX) and observed the recovery process of sympathetic nerve. He found that the sympathetic nerve disappeared after the surgery at first but new sympathetic nerve appeared on the mesenteric arteries and veins again 5 weeks later, after the surgery. Their observation was similar to ours and our result of glyoxylic acid staining also showed that the sympathetic nerve of femoral artery was almost invisible on the 7th and 14th postoperative day after being treated with 6-OHDA, and a few of new sympathetic nerves appeared again on the 28th day.

TH exists in the cytoplasm of adrenergic nerve cells and NPY is secreted by sympathetic nerve. Both TH and NPY are used as specific markers of sympathetic nerve in many studies [14]. To further identify whether our denervation model was successful and how long the inhibition effect lasted, the protein expression of the TH and NPY was observed in our study. The western blotting results of TH and NPY showed that after the treatment of 6-OHDA, the expression of both proteins had the same tendency. The protein quantity was very little on postoperative day 7 and day 14 and increased a little on day 28. The western blotting result was consistent with the observation of glyoxylic acid staining and both proved that 6-OHDA could be used to inhibit the sympathetic nerve from artery system temporarily.

## 5. Conclusion

Our study provided an effective method to inhibit the sympathetic nerve from the arterial vessel wall temporarily by using 6-OHDA* in situ*. We established a rabbit model of temporary local denervation of sympathetic nerve* in situ* and this model could help us to find out the relationship between neurotization and vascularization in the process of bone regeneration.
